# Changes of Gambling Patterns during COVID-19 in Sweden, and Potential for Preventive Policy Changes. A Second Look Nine Months into the Pandemic

**DOI:** 10.3390/ijerph18052342

**Published:** 2021-02-27

**Authors:** Anders Håkansson, Carolina Widinghoff

**Affiliations:** 1 Department of Clinical Sciences Lund, Psychiatry, Faculty of Medicine, Lund University, S-22100 Lund, Sweden; carolina.widinghoff@med.lu.se; 2 Region Skåne, Malmö Addiction Center, Clinical Research Unit, S-20502 Malmö, Sweden

**Keywords:** COVID-19, pandemic, gambling disorder, problem gambling, legislation, policy

## Abstract

Gambling has been suggested as one of the potential mental health consequences of the COVID-19 pandemic. In earlier self-report studies, increased gambling has been reported by a limited proportion of respondents characterized with a high degree of problem gambling. The present study, carried out with the same methodology and in the same geographical setting, around seven months later in the pandemic, aimed to repeat and to extend the understanding of potential gambling changes in the population during COVID-19. An anonymous sample of web panel members was assessed, altogether 2029 individuals (52% women, 10% moderate-risk or problem gamblers). Results indicated that 6% reported increased gambling, and 4% reported decreased gambling during the pandemic. Having increased gambling was associated with more severe gambling problems (OR 2.78, 95% confidence interval 2.27–3.40), increased alcohol consumption (OR 2.92, 1.71–4.98), and psychological distress (OR 3.38, 1.83–6.23). In the group reporting increased gambling during COVID-19, moderate-risk/problem gambling was very common (62%). Recent governmental policy interventions in the area were known to a minority (30%) of respondents, but awareness of the regulations was markedly more common in individuals with at least moderate-risk gambling (56%) and in self-excluders (78%). Reporting of any perceived influence from policy changes was low (3%), and divided between those reporting an increasing and decreasing effect, respectively. Increased gambling may be a consequence of COVID-19-related changes in everyday lives of individuals with problematic gambling patterns. Thus, a vulnerable group demonstrates higher rates of gambling migration and psychosocial problems, and may require particular attention in screening and treatment contexts, and further scientific evaluations.

## 1. Introduction

As one of the potential psychological and social consequences of the COVID-19 pandemic, authors have raised concerns that gambling behaviors may change and that problem gambling may increase [1,2]. Authors have highlighted a number of reasons to suspect a link between COVID-19-related changes in society, and a possible increase in gambling problems. These may include the removal of protective factors including structured everyday life [1], or boredom and negative affect during the pandemic [3], as well as financial deprivation [4].

Consequences on everyday lives and mental suffering have been described using the theory of terror management, underlining the dramatic impact resulting from the presence of COVID-19 in a society [5]. Psychological reactions to the pandemic, and to the fear of disease and mortality, have been hypothesized to have a broad impact on human behavior, including consumer behavior [6]. Everyday lives of many people have been substantially altered, with a high degree of home schooling for school children and students [7], also with likely negative effects on young people and their families [8]. Likewise, COVID-19-related restriction and changes in many people’s lives have led to significant insecurity of one’s job situation and fear of unemployment and financial problems [9].

Early during the pandemic, researchers in the area of mental health expressed concerns that COVID-19 may have serious consequences on mental health, i.e., consequences far beyond the acute physical danger of the disease [10,11], and that treatment systems for mental health issues may need to adapt in order to meet mental health patients during COVID-19 [12]. This also has included concerns that addictive disorders may increase or that its treatment may need substantial adaptation during the pandemic [13]. During the early phases of the pandemic, a limited number of research studies assessed potential impact of the COVID-19 pandemic on gambling behavior and on problem gambling. In Sweden, in an online survey addressing web panel members from the general population in late April and early May, around 4% of the respondents reported a subjective increase in their gambling during the COVID-19 pandemic in Sweden. This proportion of the population was smaller than the proportion of respondents reporting decreased gambling. The fulfillment of at least moderate-risk gambling on a well-established assessment instrument, measuring past-year gambling problems, was strongly associated with the reporting of increased gambling during the pandemic. This study was carried out during a period when virtually no major sports events took place in Sweden and other parts of the world [14], such that the opportunities for sports betting were reduced to a minimum related for example to the very few soccer leagues still running. In that situation, the self-reported increase in some other gambling types, due to the reduction of sports betting available, was modest, but again showed that individuals reporting such a migration in gambling were moderate-risk or problem gamblers in an overwhelming number of cases in this subgroup [15]. One further general population web survey, carried out in early May in only past-year online gamblers, confirmed the picture of land-based gambling being reduced during the pandemic, a possible increase in horse race betting while other sports were unavailable, and the fact that individuals who maintained gambling even during times of reduced gambling opportunities display a higher level of gambling problems [16]. The same picture, of a temporary increase in horse race betting and a decrease in traditional land-based betting, was shown in an analysis of revenue-based taxations of gambling operators in the same setting. From that study, financial activity in gambling operators still maintained a relatively unaffected level even when sports betting was very rare [17]. A different analysis from the same setting, studying ecological data of gamblers’ activity in gambling operators on the overall Swedish gambling market, demonstrated modest effects on gambling in Sweden during the pandemic, mainly indicating a decrease rather than the opposite [3]. In Australia, during the reduction of physical gambling opportunities, overall gambling did not demonstrate an increase, also not in the online setting, and individuals with the highest degree of gambling problems were not more likely to increase their gambling [18]. In contrast, a study carried out during physical COVID-19-related restrictions in Ontario, Canada, demonstrated some migration from land-based to online gambling, and a number of risk factors, such as anxiety, depression, being influenced to gamble due to COVID-19 and gambling under the influence of cannabis or alcohol were associated with high-risk gambling months into the pandemic in 2020 [4].

However, during later phases of 2020, after a decrease in the burden of disease from COVID-19 during the summer months, a marked surge in COVID-19 cases starting in October 2020, in Sweden as in many other countries [19]. Thus, restrictions on society were therefore prolonged or reinforced, in order to prevent further spread of the virus. While the changes in the gambling market in April and May 2020, involved a total close-down of professional sports and the physical state-owned casinos, the latter remained closed throughout the summer and autumn, whereas availability of sports betting largely was largely normalized early in the summer, as major sports events, although with limited audience present, reopened and again became available to sports bettors.

Policy makers in several settings have taken action in order to prevent a potential impact of COVID-19 on problem gambling. This includes Sweden, which passed a law introducing temporary COVID-19-related special regulations on gambling from July 2020, currently in use at least up to June, 2021. These temporary regulations include a mandatory limit-setting for time spent in online casino gambling and in land-based electronic gambling machines, as well as a limit of deposits in these two gambling types (a limit of SEK 5000 weekly, corresponding to approximately EUR 900 weekly). In addition, the welcome bonuses typically offered by commercial gambling operators are limited to a sum of SEK 100 (around EUR 9) according to the same regulations [20]. Hitherto, no scientific evaluation of the present regulations is available. Likewise, temporary policy interventions were also decided in other settings, such as total of partial bans against online gambling or gambling advertising, or deposit limits, such as in Spain, the United Kingdom, and Latvia [21,22,23]. No scientific evaluations of these policy changes have been made.

Early documentation of the present gambling market during COVID-19 demonstrated somewhat expected changes in gambling market activity with a decline mainly in sports-related and land-based gambling, and that a small percentage of the population reported having increased their gambling and a somewhat higher percentage reporting some decrease. However, beyond the most apparent changes occurring during sports lock-down in the spring of 2020, it is unclear whether such potential changes may remain later during the pandemic. In addition, scientific follow-up of policy changes is hitherto lacking. For these reasons, the present study—using virtually the same methodology and the same way of addressing the general population as in the first survey in the setting [6]—aimed to study (1) self-reported changes in gambling behavior during the COVID-19 pandemic in Sweden, and reasons for change in gambling behavior, (2) potential risk factors of increased gambling during the pandemic, including gambling patterns, problem gambling severity, psychological distress and a number of sociodemographic characteristics, (3) and the self-reported awareness and effectiveness of the temporary COVID-19-related gambling regulations in Sweden.

## 2. Materials and Methods

### 2.1. Setting

As described previously [15,16], the COVID-19 pandemic reached Sweden with a first confirmed case in late January, and with the onset of governmental restrictions developing during a few days in mid-March. With respect to sports events and societal changes with a potential impact on the gambling market, a substantial decrease of sports-related gambling opportunities occurred in mid-March, and the major state-owned land-based casinos closed on April 1. Sports gambling opportunities can be considered to have been normalized gradually from mid-May. During these COVID-19-related changes to the gambling market during the spring of 2020, and possibly reflecting the decrease in sports events, the major horse betting operator’s revenue in the gambling market in Sweden increased markedly during April and May, after which it decreased in June to more normal levels [17].

Due to the substantial surge in COVID-19 spread during the autumn of 2020, new regional restrictions were decided for one Swedish region on October 20, and in a second region on October 27, and in early and mid-November, virtually all other Swedish regions had introduced substantial restrictions. However, despite severe impact on sports athletes and specific teams in many sports [14], sports betting remained highly available, leaving few new objective changes to the gambling market from this surge. Land-based state-owned casinos still remained closed.

### 2.2. Procedures

The present study was a cross-sectional, general population web survey, distributed online to members of a voluntary web panel of a market survey company (Userneeds, Copenhagen, Denmark). Userneeds operates in a number of countries including Sweden, and provides a web panel of individuals for market surveys and similar. The electronic survey administered in the present study was designed in collaboration with Patient Information Broker AB (PIB AB) and I-Mind Consulting AB. The survey was sent to web panel members of Userneeds in order to fill representative age and gender groups of members, until 2000 responses were collected. Individuals in the web panel received a short electronic message saying that they had a new survey to consider, and that it addressed gambling and COVID-19. When clicking on that message, potential participants viewed a structured research information and the possibility to provide informed consent electronically. In case of providing informed consent, the individual was referred to the actual survey. Survey responses were transferred to I-Mind Consulting for the creation of a study database, in a system making it technically impossible to back-trace IP addresses or geographical location of the respondent. Age and gender distribution was followed daily in order to achieve a 50-50 gender distribution, and an age distribution closely comparable to that of the survey addressing gambling and COVID-19 with the same methodology, in April–May 2020 [15], and which in turn aimed to resemble the age distribution of the general population. The data collection of the present study was carried out from November 20 to November 29.

Study participants received remuneration as part of the reward system of Userneeds and in line with that company’s policy for other surveys. This reward consists of credit points in a reward system where these credits can be transferred into gifts, and where the value of credits associated with the present survey can be estimated to around EUR 1.

### 2.3. Participants

As stated above, potential study participants were web panel members of Userneeds, who voluntarily provided informed consent to participate. Minimum age was 18 years (which is also the legal gambling age in Sweden), with no maximum age, but with an age group distribution aimed to resemble the April/May study using the same design.

### 2.4. Measures

The survey was discussed and created by the two authors in collaboration. Apart from structured instruments previously known from the literature (see below), other items, including their response options, were created for the present and unforeseen situation of COVID-19, and inspired by the research group’s previous studies in the area [15,16,24], impressions gathered from clinical work in a regional specialized gambling disorder facility [25] and by details in the current COVID-19-specific gambling regulation in the country [20]. Large parts of the present online survey were identical to the one used in the study in April/May 2020 [15]. Given the format of a quantitative web survey, close-ended options were used for all study items. These measures include a number of items related to gambling and potential life-style changes during the COVID-19 pandemic in Sweden:

- The 9-item survey tool Problem Gambling Severity Index (PGSI [26]). The PGSI is a well-established instrument for the measure of gambling problems in different levels of severity, and has been used also in previous online surveys in the present setting [15,16,24]. A Cronbach-alpha of the instrument of 0.77 has been reported [27].
- Dichotomous questions about past-year use of each type of gambling (online casino, online sport betting, land-based sports betting, online horse betting, land-based horse betting, online lotteries, land-based lotteries, online poker, land-based electronic gambling machines, online bingo). Gambling types included were the ones typically occurring in previous online surveys in the same setting [15,16,24].
- Questions about whether participants perceived that during the pandemic, they had changed the amount of time spent at home (much more time, slightly more time, unchanged, or less time at home), their alcohol consumption (increased, decreased, unchanged, or does not drink alcohol, neither now nor prior to the pandemic), and their gambling (increased, decreased, unchanged, or does not gamble, neither now nor prior to the pandemic). Questions were asked about how each gambling type (online casino, online sport betting, land-based sports betting, online horse betting, land-based horse betting, online lotteries, land-based lotteries, land-based electronic gambling machines) had changed during the pandemic (increased, decreased, unchanged, does not use this type of gambling). All these items were worded in the same way as in the previous study [15].
- History of self-exclusion from gambling in a nationwide self-exclusion service including all licensed gambling types, available since 1 January 2019 (questions derived from a previous study in the same setting [24]).
- Psychological distress measured with the Kessler-6 [28]. This scale includes six items describing psychological symptoms of the past six months, with response alternatives ranging from ‘never’ to ‘all the time’, and numbered from 0 to 4, with a total possible score of 24. As in the previous study, five points or more was considered to represent at least moderate psychological distress [15]. The Chronbach-alpha of the K6 instrument has been reported to be 0.89 [29].
- Sociodemographic variables (age in age groups, gender, income in intervals, living conditions, and occupation).

In addition to the survey used in April/May 2020 [15], the present survey was updated regarding the following items:

- If an individual responded that gambling had increased, or decreased, a following question was asked about why (because of changed gambling opportunities in the market, because I am feeling psychologically worse during COVID-19, because of a changed everyday life in COVID-19, for financial reasons because I need to make more money or cannot afford gambling, or other). Response options listed in the survey were—due to the novelty of the research topic—not derived from a particular source, but inspired from the current debate and from the clinical settings [25] to which the authors are affiliated.
- A multiple-choice question asking about which gambling types (or none) the respondent had gambled for the first time ever during the COVID-19 pandemic. Again, gambling types typically occurring in previous studies [15,16,24] were included.
- A brief description of the current temporary gambling legislation during the COVID-19 [20], and questions about whether the respondent had heard about this, and whether she/he perceived that her/his gambling had changed because of it (decreased, increased, unchanged, does not gamble on these gambling types, and do not know). In addition, one question assessed whether the respondent had used deposit limits or time limits for the concerned gambling types since these regulations were introduced.
- Individuals who reported a history of self-exclusion were asked with a following question about the longest time period chosen for self-exclusion (1, 3, 6, or 12 months [24]).
- Among the questions about gambling habits during COVID-19, land-based casino gambling was omitted, as this gambling type was shown to be reduced by considerably more respondents than those who had increased it in the previous survey [15], and most likely due to the fact that land-based casinos were still closed during the study period [17].

A total of 2029 respondents had complete data. Among them, 37 subjects had missing data for one or several Kessler-6 items. Among them, 16 subjects had a score of five or more for available items, and were classified as having psychological distress, whereas one person had one item missing and a score of zero on all remaining items, and was therefore classified as not having psychological distress. The remaining 20 individuals were excluded from analyses of psychological distress.

### 2.5. Ethical Considerations

The present survey did not collect any data which can directly or indirectly be identified with a specific person. While Userneeds has knowledge about the identity of its web panel members, their survey responses were not sent to Userneeds. Both PIB AB and I-Mind Consulting AB, responsible for the data management, and the researchers, were completely unaware of the identity of study respondents.

With respect to the previous survey study carried out in April/May 2020, the Swedish ethics review board handled the research group application with a decision that the study did not require ethical permission as it did not include sensitive data which could be linked to specific identified individuals [15]. The present survey used an identical methodology for the recruitment, using the same web panel provider which was addressed in the same way. Additionally, in large parts, the survey was identical to the survey used in April/May 2020. In addition to minor omissions of study questions, new items were added asking similar questions about gambling types used for the first time during COVID-19, and in addition the questions assessing knowledge about and possible impact of the temporary COVID-19 gambling legislation in Sweden. Thus, based on the ethical board’s decision that the previous did not require ethical permission, and on the identical data collection method used here, no ethical permission was required by Swedish law.

### 2.6. Statistical Methods

The study analyzed variables associated with having increased gambling during COVID-19, and variables associated with awareness of the current COVID-19 gambling regulations. Other analyses were mainly descriptive. Associations with increased gambling were calculated both for the full sample, and when excluding all individuals who reported not having gambled at all, neither now nor prior to the pandemic (nongamblers). Variables which were significantly associated with increased gambling in the bivariate analyses were thereafter entered simultaneously in a logistic regression analysis. Here, however, as the vast majority of respondents reported having spent more time at home during the pandemic, this variable was not entered into the logistic regression. A sensitivity analysis, including the variable in the regression, demonstrated that apart from that association (with a very wide confidence interval), the other significant associations seen remained the same. For the analysis of awareness of the current legislation, no such logistic regression was carried out. Associations with a *p* value below 0.05 were considered significant.

## 3. Results

### 3.1. Gambling Data and COVID-19-Related Effects on Gambling Behavior

Descriptive characteristics of the sample are displayed in Table 1. Ten percent (n = 204) were at least moderate-risk gamblers, and 3% (n = 64) had ever self-excluded from the national self-exclusion service Spelpaus. Six percent (n = 114) reported gambling more during COVID-19, 4% (n = 89) reported gambling less, 54% (n = 1098) reported an unchanged gambling pattern, and the remaining 36% (n = 728) reported that they did not gamble, neither now, nor prior to the pandemic (nongamblers).

The proportion of moderate-risk/problem gamblers was 62% (n = 71) among respondents who reported increasing their gambling during COVID-19, 30% (n = 27) among those reporting decreased gambling, 9% (n = 95) among those reporting unaffected gambling, and 2% (n = 11) among nongamblers. The subsample reaching the cut-off for problem gambling constituted 35% (n = 40) of those increasing gambling, 19% (n = 17) of those decreasing their gambling, 4% (n = 47) of those reporting unchanged gambling, and below 1% (n = 6) of nongamblers. For all gambling types, the number of individuals who reported an increase was lower than the number reporting a decrease (Table 2).

In bivariate analyses, having increased gambling during COVID-19 was significantly associated with higher problem gambling severity, younger age, lower income, irregular occupation, psychological distress, a history of self-exclusion, increased time at home, increased alcohol consumption, and with a past-year history of each of the gambling types included, respectively (Table 3). In logistic regression, involving all included individuals (and where increased time spent at home was excluded due to the overwhelming majority reporting this), a self-reported increase in gambling was associated with higher problem gambling severity, increased alcohol consumption, and with psychological distress. In the subsample of gamblers (excluding nongamblers), the same variables remained independently associated with reporting increased gambling (Table 4).

Reasons for increasing or decreasing gambling during the pandemic are listed in Table 5. For changes in both directions, changes in everyday life was the most common reason reported.

### 3.2. Initiation of New Gambling Types during the COVID-19 Pandemic

A minority of respondents reported to have tried a new gambling type for the first time, after the outbreak of COVID-19: the new gambling types reported were online horse betting (3%, n = 65), online casino (3%, n = 59), online sports betting (3%, n = 53), land-based horse betting (2%, n = 44), land-based casino gambling (2%, n = 42), online bingo (2%, n = 38), land-based sports betting (2%, n = 36), online poker (1%, n = 30), and land-based electronic gambling machines (1%, n = 26). Ninety-one percent (n = 1847) reported not having tried any new gambling during COVID-19.

The proportion of moderate-risk/problem gamblers, and problem gamblers, respectively, was high among those reporting initiation during COVID-19 for several gambling types: online casino gambling (71% moderate-risk or problem gamblers, n = 42, including 54% problem gamblers, n = 32), land-based casino (71%, n = 30, 62%, n = 26), online sports betting (62%, n = 33, 47%, n =25), land-based sports betting (47%, n = 17, 42%, n = 15), online poker (57%, n = 17, 50%, n = 15), land-based electronic gambling machines (58%, n = 15, 46%, n = 12), online bingo (54%, n = 21, 33%, n = 13), online horse betting (43%, n = 28, 37%, n = 24), and land-based horse betting (45%, n = 20, 39%, n = 17). In those explicitly reporting that they had not initiated any new gambling type during the pandemic (n = 1847), the corresponding figures were 5% (n = 96) and 2% (n = 28).

### 3.3. Awareness and Experience of COVID-19-Related Gambling Regulations

Regarding the temporary COVID-19-related gambling regulations, 30% (n = 614) were aware of these regulations. Two percent (n = 32) reported feeling that these regulations had increased their gambling, 1% (n = 25) that the regulations had decreased their gambling, 30% (n = 605) reported that their gambling was not affected by the regulations, and 66% (n = 1340) reported that they were unaffected by the regulations as they do not use any of the gambling types concerned. One percent (n = 27) refused to answer or did not know. Five percent (n = 94) reported having applied the temporary deposit limits, 22% (n = 450) had not, 72% (n = 1463) were unaffected by these regulations as they do not gamble on these gambling types, and 1% (n = 22) refused to answer or did not know.

Among the 25 individuals who reported having decreased their gambling due to the new regulations, 80% (n = 20) were problem gamblers and another 12% (n = 3) were moderate-risk gamblers. Among the 32 individuals who reported having increased their gambling due to these regulations, 81% (n = 26) were problem gamblers, and another 13% (n = 4) were moderate-risk gamblers. In contrast, among individuals who denied having been affected by the regulations, the majority were either no-risk (69%, n = 418) gamblers or low-risk gamblers (13%, n = 79).

The percentage of respondents who were aware of the legislation was higher in men (37%) than in women (24%, *p* < 0.001), higher in those reporting at least moderate-risk gambling (56%) than in others (27%, *p* < 0.001), lower in those describing themselves as nongamblers both now and prior to the pandemic (21% vs. 36% *p* < 0.001), and higher in self-excluders (78%) than in others (29 %, *p* < 0.001). The highest rates of awareness of the regulations were seen among those reporting past-year online casino gambling (62% vs. 27%, *p* < 0.001) or online poker gambling (64% vs. 28%, *p* < 0.001). In the group reporting past-year land-based machine gambling, 49% reported to be aware of the regulations.

As seen in Table 6, those reporting to be aware of the COVID-19 gambling regulations were significantly more likely, for all gambling types, to report past-year gambling, and they were significantly more likely to be men, to have ever self-excluded from gambling, to be at least moderate-risk gamblers, to not report being a nongambler, and to be older, whereas they did not differ with respect to income, living alone, psychological distress, or occupation. When excluding nongamblers, 1301 individuals were included in the second analysis. Here, awareness of the legislation was significantly associated with male gender (*p* < 0.001), all types of past-year gambling (*p* < 0.001) except land-based horse betting (*p* = 0.05), history of self-exclusion (*p* < 0.001), having increased gambling during COVID-19 (*p* < 0.001), and to being at least a moderate-risk gambler (*p* < 0.001), but unrelated to psychological distress (*p* = 0.59, 11 individuals excluded), irregular occupation (*p* = 0.68), income (*p* = 0.70), age (*p* = 0.26), and living alone (*p* = 0.87).

## 4. Discussion

The present study, focusing on gambling behaviors during the COVID-19 pandemic, was carried out in November 2020, with a methodology virtually identical to the one used in a prior study earlier in the pandemic (April–May 2020). As in the previous study [15], a particularly vulnerable group appeared, presenting with a higher degree of gambling problem severity, and a self-reported increase in alcohol consumption, were independently associated with reporting increased gambling during the pandemic, and in the present study, psychological distress also became independently associated with increased gambling. In addition, the same correlates of increased gambling were seen when excluding respondents who reported not to be gamblers at all. While these associations therefore remain similar to those seen early in the pandemic, some differences were seen in comparison to the study carried out early in the pandemic; while the April/May study demonstrated a higher proportion reporting decreased gambling than the proportion reporting increased gambling, the opposite was seen here. The proportion reporting increased gambling tended to be higher than in the early study, and the proportion reporting a decrease tended to be lower than in that study [15]. In case of an increase in gambling activity in the population, this could result in a higher harmful consumption and related harms, according to the previously described total consumption model [30], and requires further evaluation.

Despite methodological challenges with estimating changes in gambling patterns over time, considering possible measurement errors and the high number of affecting variables [31], the present study still deepens the understanding of self-reported changes in gambling behavior occurring during the pandemic. A significant majority of those reporting an increase in gambling were at least moderate-risk gamblers, and many fulfilled criteria of problem gambling. Thus, the present study confirms the impression that individuals reporting an increase in gambling during the pandemic represent a group with very high degree of problems and likely need structured preventive and therapeutic interventions.

Thus, the present study strengthens the hypothesis that gambling may change in different ways in different subgroups in the population during COVID-19 [15], and that problem gamblers may be more likely to report increasing gambling habits during the pandemic. However, in contrast to this, Italian researchers reported that the pandemic was instead perceived as a relief to problem gamblers, when lockdown procedures and the reduction of sports events decreased the possibilities for gambling [32]. Additionally, the findings from the present setting are in contrast with those from Australia [8], where gamblers responding to a survey did not systematically report a higher likelihood of increasing gambling in case of gambling problems (although such a trend was seen for the subgroup with a lower degree of problem but not for the group with the highest severity scores). It can be discussed whether the characteristics of each separate gambling market may influence the effects of COVID-19; in the market studied here, Sweden, online gambling is very common [24,25], and for many gamblers, their preferred gambling types were not affected by the outbreak of COVID-19. The exception would be the weeks in March to May when sports events were cancelled, but even during this period, online horse race betting is believed to have increased, and online casino gambling, being the most common gambling type among problem gamblers in the setting, was for natural reasons unaffected by COVID-19-related restrictions. Therefore, the same type of relief as reported in the study of Donati and coworkers [32], may not be as likely in a highly online-based gambling market. Thus, it can be hypothesized that the degree of online involvement in the gambling market may lead to diverse effects from the COVID-19. Given the psychological and social consequences of COVID-19, in line with terror management theory [5], it may be likely that particularly vulnerably groups may be more likely to engage in high-risk gambling on a highly accessible gambling market. With relevance to the present setting, such a situation may be a potentially increased risk of individuals with mental distress to engage in online gambling [24]. 

In addition, the present study is the first to evaluate government interventions aiming to control gambling during the pandemic period. While the proportion reporting awareness of the special regulations was relatively low, a majority of moderate-risk/problem gamblers were aware of the legislation, and this proportion was more than twice as high in online casino or online poker gamblers than in others. Additionally, a large majority of self-excluders were aware of the regulations, demonstrating that knowledge about these regulations reached, to a markedly higher extent, people with more intense gambling habits.

Only a low proportion of respondents reported that the new legislation had any influence on their gambling, but in both the small groups reporting increased or decreased gambling as a result of the regulations, rates of gambling severity were very high; a very large majority in both these groups were at least moderate-gamblers. Thus, these findings indicate that groups with problematic gambling may be more prone to change their gambling habits. This may be seen as a similar finding to the one indicating that individuals with problem gambling were more likely to report initiation on new gambling types during the pandemic. Thus, individuals with a more intense gambling pattern may also be those reporting the more volatile patterns over time, such as in the current pandemic. Meanwhile, it should be borne in mind that the influence on gambling reported here is entirely a self-reported, subjective measure. This may be subject to both recall bias, and possibly a bias related to an individual’s own motivation for change, or attitudes to gambling and to responsible gambling measures.

The present follow-up of introduced COVID-19-related legislations is the first scientific paper to address the effects or experience of such interventions. Similar interventions were carried out in a number of European countries early in the pandemic [21,22,23]. While no previous scientific evaluations are available for comparison to the ones made here, the present findings also may be of interest to stakeholders in other settings, who may need to evaluate efforts taken against problem gambling during COVID-19.

Only few individuals started new gambling types during the pandemic. As stated above, they were markedly more likely to have gambling problems. The latter was less pronounced for horse betting. In previous studies from the present setting during the COVID-19 pandemic, there has been an indication of an increased activity in horse betting during the spring of 2020 [17], but also a trend where people with recent horse betting during the early phases of the pandemic had fewer gambling problems than several other recent gamblers in that period [16]. Thus, compared to gamblers initiating other types of gambling, people turning to horse betting may share more characteristics with gamblers without gambling problems. Price [4], studying casino gamblers in Ontario, Canada, also demonstrated that relatively few gamblers turned to online gamblers during land-based casino lockdown, and that those who did were likely to have been online gamblers already before [4]. Thus, migration towards new types of gambling are not likely to occur in broad groups in the population, but to various extents in various subgroups. Here, few previous studies are available for comparison, given the novelty of the situation the world is facing during COVID-19. Previously, during a financial crisis which hit a number of countries, Icelandic research demonstrated some changes in the types of gambling preferred; for example, increase lottery gambling was seen in individuals with financial problems during the crisis [33]. Such effects are likely difficult to compare to changes occurring during COVID-19, as previous financial crises have not involved the same type of physical lockdown procedures and the nearly total cancellation on sports events, as seen during this pandemic. Altogether, the present data confirm the previous picture that during a crisis like the present one, those who increase their gambling patterns are likely to have a markedly higher degree of problems.

An important finding was that in contrast to the similar population survey carried out early during the pandemic, the ratio of individuals reporting increased vs. decreased gambling had now been changed; the proportion reporting an increase was now larger. This may potentially have different explanations; possibly, some individuals who reported decreased gambling in the spring of 2020 may have done so only because of the shortfall of sports betting during that period, as the study was carried out when sports betting opportunities in established sports events and leagues were virtually nonexistant. In contrast, although with adaptations to the pandemic situation, sports events thereafter became active again, and major soccer leagues and many other sports events were available for betting when the present study was carried out. Still, at the time of the present study, the major land-based, state-owned casinos in major cities in Sweden were still closed, but technically, all other gambling options were available for the most of the period of COVID-19 transmission in Sweden, apart from the major sports betting events during a number of weeks in April to June 2020. Thus, while reasons for decreasing gambling may now be fewer, one more explanation could be possible. It cannot be excluded that changes in society during COVID-19 may affect gambling, including problematic gambling, in an increasing direction rather than the opposite. Here, for example, psycho-social distress, job insecurity and worry about the future may be factors leading to increased gambling, as these factors of financial insecurity and psychiatric comorbidity typically represent risk factors of problem gambling [1,3].

The results of the present study may have implications of relevance to policy making and to clinical settings in the gambling area. Policy implications may be particularly important on the current, highly available, gambling market. Parallel to the development of new technology with unlimited gambling opportunities, annual gross gambling revenues have increased steadily globally since the beginning of the 21st century. Market growth and development of large global corporations have been enabled by lack of effective policy regulations often justified by tax revenues as a ‘good cause’ [34]. Problem gambling has been shown to be associated with high societal cost [35], and cost-effective preventive efforts need to be further studied. Policy interventions may be of crucial importance, due to the fact that a very large percentage of gambling-related harm is related to a small number of gamblers [36]. Additionally, based on the theory that addiction treatment and mental health treatment may require substantial adaptations during COVID-19 [12,13], increased gambling in the group screening positive for problem gambling may require adapted outpatient treatment facilities, including a substantial shift towards telepsychiatry and other distance contacts.

Related to clinical implications, as in the previous study, it was shown that individuals reporting increased gambling during the pandemic was a particular risk group. Thus, structured screening for early detection of changing gambling behaviors may be of relevance, in clinical settings where gambling problems can be detected and/or treated, as well as in responsible gambling measures potentially applied by gambling operators. In addition, the study findings indicate some support of a generally cautious societal approach in the area, which takes into account the risk of gambling developing as a negative psychosocial consequence of the pandemic. Thus, although the effect of early policy changes cannot be proven or quantified here, the study confirms the picture of some migration between gambling types, and that individuals who do migrate to new gambling types are a particular risk group. Thus, on a highly available gambling market, individuals who are subject to migration between gambling types during this crisis are a group in need for clinical screening and treatment. Therefore, policy changes, including follow-up of their efficiency, need to focus on early detection of such behavioral changes, and should aim for harm-reducing interventions whenever such gambling changes are seen.

Implications of the study may be related to the design of preventive measures aiming to decrease harms of gambling during the times of COVID-19. From the present study, it can be concluded that the present regulations, in use only for online casino gambling and for electronic gambling machines, and with a relatively high weekly deposit limit, was in fact used by a small minority of gamblers. The policy changes aimed to address only the gambling types perceived to have the highest addictive potential, i.e., rapid, chance-based games. While this may therefore fulfill the purpose intended, it goes beyond the present study to assess whether other preventive measures would have been needed. However, it is clear that the minority reporting to have initiated new gambling types during the pandemic represent a particular risk group. Therefore, one possible implication could be to implement special motivational advice by gambling operators when individuals are discovered to change their gambling behaviors and to initiate new gambling types.

The present study has limitations. These are mainly related to the fact that the included data are all self-reported, and therefore may be influenced by a certain recall bias. In addition, as shown also in previous studies using the same methodology, online surveys addressing gambling-related issues in this web panel tend to include respondents with pronounced gambling habits, and likely online gamblers rather than individuals with an entirely land-based gambling pattern. However, gambling online is common in the present setting [25], and also represents the types of gambling most commonly displayed in televised gambling advertisements [37]. Thus, findings cannot be readily generalizable to the whole general population, as participants in the study may be more prone to engage in online gambling. In contrast, however, respondents may differ less from the general population in the present setting, where online gambling is very common overall. Moreover, the generalizability of the findings cannot be established beyond the present geographical setting. Previous literature has discussed that the geographical setting may influence gambling research findings, through factors such as sociodemographical distribution and minority representation [38]. In addition, the impact of COVID-19-related regulations in on Sweden has differed from that of many countries; policies against virus transmission leaned on public recommendations rather than on stricter regulations such as home confinement or the shutdown of businesses [39]. In contrast, the present study has the strength of having used the same methodology as the first study carried out in the same geographical setting, and therefore allows for comparisons to be made.

The survey was entirely anonymous and therefore could not address the same individuals. The proportion of women was slightly larger (52% vs. 49% in the previous study). Proportions of age groups were similar; in the present study, however, the second oldest age group was somewhat smaller (21% vs. 26% in the previous study), but the oldest age group somewhat larger (23% vs. 21% in the previous study). In both studies, younger age was associated with a higher likelihood of having increased gambling during the pandemic. Thus, it cannot be excluded that the limited different in age distribution in the older age groups may have elevated the proportion of respondents who had increased gambling in the present study.

## 5. Conclusions

In conclusion, the present study demonstrates that a limited proportion of the general population may have increased gambling during the COVID-19 crisis. Additionally, the ratio between the numbers of individuals increasing and decreasing their gambling appeared to be somewhat higher than in the early phases of the pandemic. Life-style-related changes in COVID-19 were reported as the main reason for both increasing, and decreasing, gambling during the crisis. Individuals initiating new gambling types during the pandemic represented a particular risk group, with an overwhelming majority meeting the criteria of moderate-risk or problem gambling. Gambling severity, psychological distress, and increasing alcohol consumption, characterized the group reporting increased gambling. Treatment services should aim to identify and treat patients with increasing gambling patterns during COVID-19, and adapt to the wider challenges in society caused by the pandemic, including the need to develop tele-psychiatry methods and facilitate treatment seeking for problem gambling. Additionally, in conclusion, few respondents reported a change in their gambling following the temporary COVID-19 prevention policies. Although a minority were aware of the new legislation, this was clearly related to being a gambler and to fulfill a higher degree of gambling problems.

Thus, this type of preventive policy measure may have potential for an effect in populations at risk. Previous research from the present setting also emphasizes the need of public debate to influence gambling norms [16] and encourage more people with high risk or problem gambling to seek help [40]. Today’s global gambling industry brings an extensive need of protection of the vulnerable groups, here presented by gamblers who increased their gambling during the COVID-19 crisis. There are reasons to take further policy actions, as research indicates that substantial limitations are required to have substantial effects [41] and attention should be payed to discussions concerning the ethical issues of gambling harm beyond cost-effectiveness. From a public health perspective, voices are raised to implement forceful regulatory measures at an early stage, to reduce the cases in need of emergency care [34,42]. More research, including quantified gambling data and follow-up measures, is required, in order to fully analyze the objective effect of the intervention.

## Figures and Tables

**Table 1:** Sample description. All individuals (n = 2029).

	n (%)
Age groups	
- 18–24 years	142 (7)
- 25–29 years	211 (10)
- 30–39 years	361 (18)
- 40–49 years	425 (21)
- 50–64 years	427 (21)
- 65 years and above	463 (23)
Female gender	1051 (52)
Living conditions	
- Alone without children	519 (26)
- With partner, no children	734 (36)
- With partner and children	575 (28)
- Living alone with children	115 (6)
- Living with parents	86 (4)
Monthly income	
- SEK <10,000	199 (10)
- SEK 10,000–15,000	223 (11)
- SEK 15,000–20,000	201 (10)
- SEK 20,000–25,000	232 (11)
- SEK 25,000–30,000	315 (16)
- SEK 30,000–35,000	279 (14)
- SEK 35,000–40,000	211 (10)
- SEK 40,000–45,000	135 (7)
- SEK 45,000–50,000	82 (4)
- SEK >50,000	152 (7)
Occupation	
- Working	1129 (56)
- Studying	169 (8)
- Retired	500 (25)
- Job-seeking	105 (5)
- Sick-leave	59 (3)
- Short-term pandemic-related unemployment benefit	15 (1)
- Other	52 (3)
Psychological distress	
- Kessler-6 score (median, range, inter-quartile range)	4 (0–24, 1–9) *
- Moderate psychological distress (cut-off 5 or above)	991 (49) *
Past-year gambling, any	
- online casino	191 (9)
- land-based casino	87 (4)
- sports betting online	417 (21)
- sports betting, land-based	238 (12)
- horse betting, online	418 (21)
- horse betting, land-based	225 (11)
- poker online	110 (5)
- electronic gambling machines, land-based	97 (5)
- bingo online	166 (8)
Problem gambling severity	
- no risk	1686 (83)
- low risk	139 (7)
- moderate risk	94 (5)
- problem gambling	110 (5)

* 20 cases excluded due to missing data on one or several Kessler-6 items.

**Table 2:** Self-reported COVID-19 effect on each type of gambling. All items including 2029 subjects.

	Increased, n (%)	Decreased, n (%)	Unaffected, n (%)	Do Not Gamble on This Type, n (%)
Online casino	40 (2)	58 (3)	225 (11)	1706 (84)
Sports betting online	54 (3)	98 (5)	412 (20)	1465 (72)
Sports betting land-based	26 (1)	149 (7)	408 (20)	1446 (71)
Horse betting online	72 (4)	91 (4)	432 (21)	1434 (71)
Horse betting land-based	24 (1)	163 (8)	231 (11)	1611 (79)
Lotteries online	85 (4)	91 (4)	568 (28)	1285 (63)
Lotteries land-based	70 (3)	272 (13)	1030 (51)	657 (32)
Electronic gambling machines, land-based	29 (1)	117 (6)	246 (12)	1637 (81)

**Table 3:** Comparison of respondents with increased gambling and respondents with decreased, unchanged, or no gambling.

	Increased Gambling (n = 114), n (%)	Did Not Increase Gambling (Decreased, Unchanged, or No Gambling, n = 1915), n (%)	*p* Value
Age groups - 18–24 years - 25–29 years - 30–39 years - 40–49 years - 50–64 years - 65 years and above	16 (14) 32 (28) 20 (18) 19 (17) 15 (13) 12 (11)	126 (7) 179 (9) 341 (18) 406 (21) 412 (22) 451 (24)	<0.001
Female gender	56 (49)	995 (52)	0.56
Living conditions - Alone without children	32 (28)	487 (25)	0.53
Monthly income - SEK <10,000 - SEK 10,000–15,000 - SEK 15,000–20,000 - SEK 20,000–25,000 - SEK 25,000–30,000 - SEK 30,000–35,000 - SEK 35,000–40,000 - SEK 40,000–45,000 - SEK 45,000–50,000 - SEK >50,000	18 (16) 20 (18) 4 (4) 13 (11) 20 (18) 18 (16) 4 (4) 9 (8) 4 (4) 4 (4)	181 (9) 203 (11) 197 (10) 219 (11) 295 (15) 261 (14) 207 (11) 126 (7) 78 (4) 148 (8)	0.01
Irregular occupation	21 (18)	158 (8)	<0.001
Psychological distress - Moderate psychological distress (cut-off 5 or above)	99 (87)	892 (47)	<0.001
Past-year gambling, any - online casino - land-based casino - sports betting online - sports betting, land-based - horse betting, online - horse betting, land-based - poker online - electronic gambling machines, land-based - bingo online	51 (45) 20 (18) 57 (50) 36 (32) 59 (52) 35 (31) 28 (25) 17 (15) 40 (35)	140 (7) 67 (3) 360 (19) 202 (11 359 (19) 190 (10) 82 (4) 80 (4) 126 (7)	<0.001 <0.001 <0.001 <0.001 <0.001 <0.001 <0.001 <0.001 <0.001
Moderate-risk/problem gambling	71 (62)	133 (7)	<0.001
Severity - no risk - low risk - moderate risk - problem	31 (27) 12 (11) 31 (27) 40 (35)	1655 (86) 127 (7) 63 (3) 70 (4)	<0.001 *
Ever self-excluded	26 (23)	38 (2)	<0.001
Time at home - less - slightly more - much more - unchanged	0 (0) 32 (28) 80 (70) 2 (2)	13 (1) 692 (36) 915 (48) 295 (15)	<0.001 **
Alcohol consumption - less - more - unchanged - does not drink	20 (18) 34 (30) 47 (41) 13 (11)	321 (17) 152 (8) 1116 (58) 326 (17)	<0.001 ***

* Chi-square linear-by-linear analysis. ** analyzed for more time (slightly more and much more time) vs. other options. *** analyzed for increased consumption vs. all other options.

**Table 4:** Variables associated with self-reported increase in gambling. Logistic regressions, including all individuals with complete data (n = 1996), and all individuals after the exclusion of those reporting to be nongamblers.

Whole Sample with Complete Data (n = 1996)	OR	95% Confidence Interval	Whole Sample after Excluding Individuals Who Denied Gambling Now and Prior to COVID-19 (n = 1281). OR	95% Confidence Interval
Gambling severity (increasing level)	2.78	2.27–3.40 *	2.25	1.83–2.78 *
Older age	1.07	0.91–1.26	1.01	0.86–1.20
Irregular occupation	1.53	0.82–2.84	1.34	0.71–2.50
Income	0.99	0.90–1.09	0.96	0.88-1.06
Increased alcohol consumption	2.92	1.71–4.98 *	2.97	1.72–5.12 *
Ever self-excluded	1.37	0.68–2.79	1.31	0.64–2.67
Psychological distress	3.38	1.83–6.23 *	3.57	1.93–6.58 *

* significant association (*p* < 0.05).

**Table 5:** Reasons for increasing (n = 114) or decreasing (n = 89) gambling during COVID-19. The two groups are mutually exclusive. More than one option can be selected, and therefore figures within each group do not sum up to 100%. n (%).

Reasons for increasing gambling during COVID-19 (n = 114) - changes in the gambling opportunities offered - because I have been feeling worse psychologically during COVID-19 - because my everyday life has been changed - for financial reasons, because I need to make more money during COVID-19 - other reasons	21 (18) 30 (26) 67 (59) 29 (25) 15 (13)
Reasons for decreasing gambling during COVID-19 (n = 89) - changes in the gambling opportunities offered - because I have been feeling worse psychologically during COVID-19 - because my everyday life has been changed - for financial reasons, because I cannot afford gambling during COVID-19 - other reasons	14 (16) 9 (10) 39 (44) 27 (30) 24 (27)

**Table 6:** Variables associated with awareness of COVID-19 legislation for gambling.

	Aware of COVID-19-Related Gambling Legislation (n = 614), n (%)	Unaware of COVID-19-Related Gambling Legislation (n = 1415), n (%)	*p* Value
Age groups - 18–24 years - 25–29 years - 30–39 years - 40–49 years - 50–64 years - 65 years and above	59 (10) 54 (9) 83 (14) 101 (16) 119 (19) 198 (32)	83 (6) 157 (11) 278 (20) 324 (23) 308 (22) 265 (19)	0.001 *
Female gender	250 (41)	801 (57)	<0.001
Living conditions - Alone without children	165 (27)	354 (25)	0.38
Monthly income - SEK <10,000 - SEK 10,000–15,000 - SEK 15,000–20,000 - SEK 20,000–25,000 - SEK 25,000–30,000 - SEK 30,000–35,000 - SEK 35,000–40,000 - SEK 40,000–45,000 - SEK 45,000–50,000 - SEK >50,000	54 (9) 70 (11) 59 (10) 88 (14) 81 (13) 79 (13) 65 (11) 45 (7) 28 (5) 45 (7)	145 (10) 153 (11) 142 (10) 144 (10) 234 (17) 200 (14) 146 (10) 90 (6) 54 (4) 107 (8)	0.71 *
Irregular occupation	53 (9)	126 (9)	0.84
Psychological distress ** - Moderate psychological distress (cut-off 5 or above)	285 (47)	706 (50)	0.15
Past-year gambling, any - online casino - land-based casino - sports betting online - sports betting, land-based - horse betting, online - horse betting, land-based - poker online - electronic gambling machines, land-based - bingo online	119 (19) 46 (7) 203 (33) 109 (18) 193 (31) 90 (15) 70 (11) 48 (8) 86 (14)	72 (5) 41 (3) 214 (15) 129 (9) 225 (16) 135 (10) 40 (3) 49 (3) 80 (6)	<0.001 <0.001 <0.001 <0.001 <0.001 <0.001 <0.001 <0.001 <0.001
Moderate-risk/problem gambling	115 (19)	89 (6)	<0.001
Ever self-excluded	50 (8)	14 (1)	<0.001
Increased gambling during COVID-19	57 (9)	57 (4)	<0.001
Nongambler (reported no gambling now or previously, in the COVID-19 and gambling item)	151 (25)	577 (41)	<0.001

* Chi-square linear-by-linear analysis. ** Data missing for 20 individuals.

## Data Availability

Data can be made available upon request to the first author.
